# Prevalence of Antibiotic Prescribing for Acute Respiratory Tract Infection in Telehealth Versus Face-to-Face Consultations: Cross-Sectional Analysis of General Practice Registrars’ Clinical Practice

**DOI:** 10.2196/60831

**Published:** 2025-03-13

**Authors:** Yu Gao, Parker Magin, Amanda Tapley, Elizabeth Holliday, Jason Dizon, Katie Fisher, Mieke van Driel, Joshua S Davis, Andrew Davey, Anna Ralston, Alison Fielding, Dominica Moad, Katie Mulquiney, Lisa Clarke, Alexandria Turner

**Affiliations:** 1 GP Training Research Royal Australian College of General Practitioners Mayfield West Australia; 2 School of Medicine and Public Health University of Newcastle Callaghan Australia; 3 School of Population Health University of New South Wales Kensington Australia; 4 Clinical Research Design IT and Statistical Support Unit Hunter Medical Research Institute New Lambton Heights Australia; 5 General Practice Clinical Unit Faculty of Medicine The University of Queensland Herston Australia; 6 GP Training Medical Education Royal Australian College of General Practitioners Hobart Australia

**Keywords:** antimicrobial resistance, antibiotics stewardship, telehealth, general practice, registrars, acute respiratory tract infection, antibiotics, prescription, respiratory tract infection, RTIs, Australia, consultations, teleconsultation, teleconsult, bronchitis, sore throat, acute otitis, sinusitis, in-consultation, upper respiratory tract infection

## Abstract

**Background:**

Antimicrobial resistance is a global threat. Australia has high antibiotic prescribing rates with the majority of antibiotics prescribed by general practitioners (GPs) for self-limiting acute respiratory tract infection (ARTIs). Australian GP trainees’ (registrars’) prescribing for ARTIs may have been affected by the introduction of remunerated telehealth consultations in 2020. Understanding of the impact of telehealth on antibiotic stewardship may inform registrar educational programs.

**Objective:**

This study aimed to compare the prevalence of antibiotic prescribing by GP registrars in telehealth versus face-to-face (F2F) consultations for common cold (upper respiratory tract infection [URTI]), bronchitis, sore throat, acute otitis media, and sinusitis.

**Methods:**

A cross-sectional analysis of data from the Registrar Clinical Encounters in Training (ReCEnT) study, a multicenter inception cohort study of registrars’ in-consultation clinical and educational experiences. Analysis used univariable and multivariable logistic regression using 2020-2023 ReCEnT data. The outcome variable was “antibiotic prescribed” for new presentations of URTI, acute sore throat, acute bronchitis, acute sinusitis, and acute otitis media. The study factor was consultation type (telehealth or F2F).

**Results:**

A total of 2392 registrars participated (response rate=93.4%). The proportions of diagnoses that were managed via telehealth were 25% (5283/21384) overall, 19% (641/3327) for acute sore throat, 29% (3733/12773) for URTI, 21% (364/1772), for acute bronchitis, 4.1% (72/1758) for acute otitis media, and 27% (473/1754) for acute sinusitis. Antibiotics were prescribed for 51% (1685/3327) of sore throat diagnoses, 6.9% (880/12773) of URTI diagnoses, 64% (1140/1772) of bronchitis diagnoses, 61% (1067/1754) of sinusitis diagnoses, and 73% (1278/1758) of otitis media diagnoses. On multivariable analysis, antibiotics were less often prescribed in telehealth than F2F consultations for sore throat (adjusted odds ratio [OR] 0.69, 95% CI 0.55-0.86; *P*=.001), URTI (adjusted OR 0.64, 95% CI 0.51-0.81; *P*<.001), and otitis media (adjusted OR 0.47, 95% CI 0.26-0.84; *P*=.01). There were no significant differences for acute bronchitis (adjusted OR 1.07, 95% CI 0.79-1.45; *P*=.66) or acute sinusitis (adjusted OR 1, 95% CI 0.76-1.32; *P*=.99).

**Conclusions:**

GP registrars are less likely to prescribe antibiotics for sore throat, URTI, and otitis media when seeing patients by telehealth versus F2F. Understanding the reason for this difference is essential to help guide educational efforts aimed at decreasing antibiotic prescribing by GPs for conditions such as ARTIs where they are of little to no benefit. There was no evidence in this study that telehealth consultations were associated with greater registrar antibiotic prescribing for ARTIs. Therefore, there is no deleterious effect on antibiotic stewardship.

## Introduction

Antimicrobial resistance is a major threat to global health [1]. Australia’s antibiotic prescribing rates are much higher than countries of similar socioeconomic status. In 2021, the rate of Australian community antimicrobial use was 17.5 defined daily doses (DDD) per 1000 inhabitants per day, compared with England (15.9 DDD), Canada (11.4 DDD), and the Netherlands (7.9 DDD) [2]. The majority of antibiotics in Australia are prescribed in general practice (family practice). Inappropriate prescribing of antibiotics in outpatient settings, especially in primary care or general practice, is a major driver of antimicrobial resistance [3]. Therefore, antibiotic stewardship in general practice is vital.

In 2022, overall 36.6% of Australians were prescribed at least one antibiotic in the community [2]. The most common reason for prescription of antibiotics in general practice is nonpneumonia, self-limiting acute respiratory tract infections (ARTIs) [4]. ARTIs (common cold [upper respiratory tract infection {URTI}], acute bronchitis, acute sore throat, acute otitis media, and acute sinusitis) are not generally recommended to be treated with antibiotics, irrespective of symptom severity (in the case of URTI and bronchitis) [2,5-7]. Established Australian general practitioners (GPs) have been found to prescribe antibiotics for ARTIs at a higher rate than European GPs [2,8].

Vocational trainees in general practice are a group of particular interest regarding antibiotic stewardship. Trainees are developing and establishing patterns of practice that may be stable over time [9-11], including antibiotic prescribing [12]. In Australia, GP trainees (“GP registrars”) comprised 16% of the GP workforce by headcount in 2022-2023 [13]. They have lower antibiotic prescribing rates for ARTIs compared with established Australian GPs [14]. In 2019, established GPs prescribed antibiotics in 36% of acute URTI and 82% of bronchitis cases, whereas registrars prescribed antibiotics in 12% of acute URTI and 72% of bronchitis cases. A similar trend exists for Australian GPs and registrars’ prescribing for sore throat, otitis media, and sinusitis [15,16].

Furthermore, in longitudinal analyses between 2010 and 2019, registrars’ prescribing for URTI, acute bronchitis, sore throat, sinusitis, and otitis media reduced significantly [16,17]. URTI prescribing decreased from 24% of presentations in 2010 to 12% in 2019, acute bronchitis or bronchiolitis prescribing from 84% to 72%, sore throat from 76% to 60%, otitis media from 88% to 77%, and sinusitis from 84% to 66% [16,17]. While these temporal trends are mirrored in both established GP and registrar populations [15-17] and despite lower prescribing rates than established GPs, registrars’ antibiotic prescribing for most ARTIs still exceeds international benchmarks [5,8].

Due to community lockdown and implementation of strict infection prevention during the COVID-19 pandemic, remuneration for Australian GP telehealth consultations was introduced (via the Medicare Benefits Schedule of medical services subsidized by the Australian Government [18]). In the second quarter of 2020, overall 36% of GP consultations in Australia were conducted via telehealth and it is now an established mode of consultation in Australian general practice [19].

Antibiotic prescribing may be affected by consultation modality, that is, remote telehealth consultations as compared with face-to-face (F2F) consultations. It is plausible that clinical uncertainty related to reduced diagnostic confidence consequent upon limited ability to examine patients might drive inappropriate antibiotic prescribing [20,21]. This has implications for antibiotic stewardship. A systemic review and meta-analysis reported that antibiotics were more highly prescribed in telehealth consultations for otitis media and pharyngitis. However, no difference was found in prescribing between modalities for sinusitis, upper respiratory infections, or urinary tract infections [22]. In another systematic review and meta-analysis of 13 studies, antibiotic prescribing for respiratory, urinary, or skin and soft tissue infections in telehealth compared with F2F consultations varied depending on infection type [23]. When the 10 observational studies were pooled, there was significantly less prescribing for sinusitis in telehealth consultations and significantly more prescribing for otitis media in telehealth consultations. Pharyngitis, conjunctivitis, and urinary tract infections had nonsignificantly higher antibiotic prescribing rates in telehealth consultations. There was also no significant difference in prescribing for bronchitis between the 2 modalities [23]. However, the methodological quality of these studies indicates that more research is needed into the effect of consultation modality on antibiotic prescribing rates.

A further systematic review found that studies (published before February 2020) of remote prescribing (telephone, video, internet-based, and text-based consultations) of antibiotics for respiratory tract disease, compared with F2F consultations, had similarly conflicting results [24].

A qualitative study of Australian GP registrars and their clinical supervisors found concerns regarding impaired diagnostic capacity for ARTIs during telehealth consultations and suggested that this could lead to organizing further in-person assessment (arrangement of a F2F follow-up appointment to confirm the diagnosis) or to overprescribing of antibiotics [21]. Overall, there remains a large evidence gap relating to the effects of consultation modality on antibiotic prescribing and the potential reasons for this effect.

The context of, and impetus for, the introduction remunerated telehealth in Australia was the COVID-19 pandemic. An interrupted time-series analysis in England found the overall volume of prescribing of antibiotics primarily used for ARTIs increased between national lockdown in March 2020 and May 2022 [25]. Some Australian studies have found that there was a substantial decrease in dispensing of antimicrobials in Australia in 2020 [2], especially for antibiotics commonly prescribed for ARTIs [26]. A retrospective study also found that the proportion of respiratory tract infections (not further defined) for which antibiotics were prescribed via telehealth was lower than that of F2F consultations at an early stage of the pandemic. However, this percentage progressively increased over time, and by the end of 2021 was almost equivalent to that of F2F consultations [27].

Therefore, there is still some uncertainty regarding whether consultation modality affects antibiotic prescribing rates in primary care settings. GP registrars are a physician demographic of singular interest in this aspect of antibiotic stewardship. They are in a formative stage of their careers when practice patterns are being formed, and once established, GPs may continue to prescribe antibiotics [11,12]. A particular cause for concern related to telehealth during this formative practice period is that clinical uncertainty may drive registrars’ inappropriate antibiotic prescribing [20,21]. Given constraints on important diagnostic components (physical examination) during telehealth, it is plausible that widespread uptake of telehealth may be problematic for registrars’ antibiotic stewardship.

To the best of our knowledge, no studies have been conducted on the prevalence of GP registrars’ or trainees’ antibiotic prescribing for respiratory tract infections through telehealth. Understanding antibiotic prescribing patterns in telehealth versus F2F consultations will help inform GP registrar education and training in antimicrobial stewardship.

This study aimed to address this evidence gap by estimating the prevalence of antibiotic prescribing by GP registrars for URTI, bronchitis, sore throat, acute otitis media, and sinusitis during telehealth as compared with F2F consultations. We hypothesized that registrars would prescribe antibiotics in a greater proportion of telehealth consultations for URTI, bronchitis, sore throat, acute otitis media, and sinusitis than prescribed in F2F consultations.

## Methods

### Study Design

This was a cross-sectional analysis nested within the Registrar Clinical Encounters in Training (ReCEnT) study.

### ReCEnT Setting and Participants

ReCEnT is a multicenter inception cohort study of Australian GP registrars’ in-consultation clinical and educational experience. The detailed methodology is published elsewhere [28]. ReCEnT is a routine part of the educational program of registrars in participating training regions. Registrars may also elect to provide informed consent for the data to be used for research purposes. During the period reported here, all registrars training in New South Wales, Tasmania, and eastern Victoria (approximately 43% of Australian GP registrars) [29] were eligible for participation.

### ReCEnT Data Collection

In ReCEnT, each registrar electronically records details of 60 consecutive consultations, once in each of their first 3 general practice training terms [28]. At the beginning of each collection period, registrars provide demographics and practice data via a questionnaire. In each of the 60 individual consecutive consultations, patient demographics, diagnosis formulations, and clinical and educational actions are recorded. Data are recorded via a dedicated internet-based portal.

For the analyses reported here, data from 7 six-monthly data collection rounds between 2020 and 2023 (following the onset of COVID-19 pandemic with consequent expanded telehealth remuneration for GPs) were used.

### Outcome and Study Factor

The outcome factor was “antibiotics prescribed” (yes or no). This was determined using the Anatomical Therapeutic Chemical Classification J01 [30]. The study factor (independent variable of interest) was “consultation modality” (F2F or telehealth). “Telehealth” included both telephone and videoconference consultations.

### Independent Variables

Analyses were adjusted for a large range of independent covariates. These included registrar, patient, practice, and consultation variables. The registrar variables were age, gender, training term, full-time, or part-time training status, if qualified as doctor in Australia, and if the registrar had worked at the practice previously.

Patient variables were age group specific to the ARTI problem type (refer to Table S1 in Multimedia Appendix 1), gender, Aboriginal and/or Torres Strait Islander status, and whether the patient was new to the practice or to the registrar.

Practice variables were training region, rurality determined by the Australian Standard Geographical Classification [31], practice size (practices with <6 full-time equivalent doctors are considered “small practices”), socioeconomic status of the practice location (Socioeconomic Index for Areas-Index of Relative Social Disadvantage) [32], and billing policy (whether the practice routinely bulk bills all patients, ie, accepts the government-provided rebate as payment in full).

Consultation content variables were consultation duration, number of diagnoses managed, and whether registrars sought assistance or information during the consultation (no information source used, supervisor called, and another source used).

Consultation action variables included whether pathology was ordered, whether imaging was ordered, whether referrals were made, whether follow-ups were arranged, and whether learning goals were generated.

### Analysis Level

Analyses were conducted at the level of individual diagnosis (rather than at the registrar or consultation level). The diagnoses were restricted to initial presentations of several self-limited and nonpneumonia ARTIs: acute bronchitis, URTI, acute otitis media, acute sore throat, and acute sinusitis. Refer to Table S1 in Multimedia Appendix 1 for the International Classification of Primary Care 2 plus codes used for these diagnoses [33].

### Statistical Methods

#### Participant Characteristics

Overall characteristics of the registrars who participated in this study were calculated at either the registrar level or registrar-round level (each individual registrar contributes up to 3 rounds of data) and reported as n (%) or mean (SD).

#### Descriptive Analyses

Total diagnoses of the conditions of interest and the proportions of these diagnoses managed by telehealth were calculated. The proportion of telehealth consultations that were undertaken via video compared with phone was calculated.

The proportion of antibiotics prescribed for all new ARTI diagnoses, and individually for new URTIs, acute bronchitis, acute sore throat, acute otitis media, and acute sinusitis, was calculated for telehealth consultations and for F2F consultations. The proportion of types of antibiotics by ARTI diagnoses and consultation modality was also calculated.

#### Main Analyses

To estimate the association between consultation modality and antibiotic prescription, univariable and multivariable logistic regression models were estimated with the outcome “antibiotic prescribed” for new cases of each of the following: URTI, acute bronchitis, acute sore throat, acute otitis media, and acute sinusitis. Models were estimated within the generalized estimating equations framework to account for repeated measures within registrars. An exchangeable working correlation structure was assumed. An augmented backward selection process was followed to select explanatory variables for inclusion in the final multivariable model for each diagnosis. Variables in the model with *P* values of >.20 were tested for removal. A variable was removed if the resulting model did not have substantively different effect sizes than the previous model (more than 10% different from its value in the previous model). Model fit was assessed using the Hosmer-Lemeshow goodness of fit test. The logistic model assumption of linearity in the log-odds for continuous variables was also checked.

Regressions modeled the log-odds that antibiotics were prescribed for a given presentation. Effects are expressed as odds ratios (ORs) with 95% CIs. Significance was declared at the conventional .05 level, with the magnitude and precision of effect estimates also used to interpret results. Analyses were programmed using STATA (version 16.0; StataCorp) and SAS (version 9.4; SAS Institute).

#### Post Hoc Analysis

In post hoc analyses, the proportion of registrars’ arrangement of follow-up appointments (with themselves or another GP at the practice) following the index consultation for each ARTI, separately for telehealth and F2F index consultations, was calculated. Differences for telehealth compared with F2F index consultations were tested with chi-square tests.

Missing data were handled using complete case analysis. No imputation was performed to address the missing data (Tables S2-S6 in Multimedia Appendix 2).

### Ethical Considerations

Ethics approval was by the University of Newcastle Human Research Ethics Committee (Reference H-2009-0323) and the RACGP National Research and Evaluation Ethics Committee (NREEC-23-0000000161). Registrars provided written informed consent for data routinely collected as part of the educational program to also be used for research purposes. All data were deidentified. No compensation was provided to participants as ReCEnT is a routine part of training.

## Results

### Participant Characteristics

A total of 2392 registrars (response rate: 93.4%) contributed data from 301,403 consultations, entailing in 425,059 diagnoses. Table 1 provides details of the characteristics of participating registrars.

**Table 1:** Registrar and practice demographics of participants.

Variables and class			Values
**Registrar characteristics (n=2392), n (%)**			
	**Registrar gender**		
		Woman	1373 (57.9)
	**Has Australian medical degree, n (%)**		
		Yes	1880 (79.6)
	Year of graduation, mean (SD)		2014.5 (4.9)
	**Pathway registrar enrolled in (general or rural), n (%)**		
		General	1557 (66)
	**Has postgraduate qualifications, n (%)**		
		Yes	642 (27.2)
	**College for which seeking fellowship, n (%)**		
		RACGP^a^	2880 (95.3)
		ACRRM^b^	70 (2.9)
		Both	10 (0.4)
**Registrar-round/practice characteristics (n=5060)**			
	Registrar age (years), mean (SD)		32.8 (5.9)
	**Registrar works part-time (rather than full-time), n (%)**		
		Yes	1169 (24.1)
	**Registrar training term, n (%)**		
		Term 1	1839 (36.3)
		Term 2	1546 (30.6)
		Term 3	1675 (33.1)
	**Practice routinely bulk bills all patients, n (%)**		
		Yes	1653 (34.1)
	**Registrar had worked at practice previously, n (%)**		
		Yes	1284 (26.5)
	**Size of practice, n (%)**		
		Small (≤5 FTE^c^ GPs^d^)	2004 (41.5)
	**Rurality of practice, n (%)**		
		Major city	2873 (56.8)
		Inner regional	1876 (37.1)
		Outer regional, remote, and very remote	308 (6.1)
	SEIFA-IRSD^e^ decile of practice, mean (SD)		5.4 (2.8)
	**SEIFA-IRSD decile of practice, n (%)**		
		Decile 1	469 (9)
		Decile 2	505 (10)
		Decile 3	532 (11)
		Decile 4	633 (13)
		Decile 5	621 (12)
		Decile 6	477 (9)
		Decile 7	538 (11)
		Decile 8	382 (8)
		Decile 9	424 (8)
		Decile 10	469 (9)

^a^RACGP: Royal Australian College of General Practitioners.

^b^ACRRM: Australian College of Rural and Remote Medicine.

^c^FTE: full-time equivalent.

^d^GP: general practitioner.

^e^SEIFA-IRSD: Socioeconomic Indexes for Areas-Index of Relative Social Disadvantage [32].

### Descriptive Findings

Overall, there were 21,384 new diagnoses of our 5 nominated conditions available for analysis. These included 3327, 12,773, 1772, 1754, and 1758 new diagnoses of sore throat, URTI, bronchitis, sinusitis, and otitis media representing 0.7%, 3%, 0.4%, 0.4%, and 0.4%, of all diagnoses, respectively.

The proportions of diagnoses managed via telehealth were as follows: ARTI, 25% (5283/21384); acute sore throat, 19% (641/3327); URTI, 29% (3733/12773); acute bronchitis, 21% (364/1772); acute otitis media; 4.1% (72/1758) and acute sinusitis; and 27% (473/1754). Of the total ARTI consultations managed via telehealth (n=5283), most were managed over the phone (video consult rate: 3.8% [201/5283]). Patient and consultation factors are given in Table 2 for telehealth versus F2F consultations.

For all ARTIs, overall, antibiotics were prescribed in 20% (1035/5283) of telehealth diagnoses compared with 31% (5015/16101) of F2F diagnoses. Refer to Table S7 in Multimedia Appendix 3 for the most commonly prescribed antibiotics for each condition. Refer to Tables S8-S12 in Multimedia Appendix 4 for the characteristics associated with prescribing antibiotics for each ARTI condition.

**Table 2:** Patient and consultation factors according to consultation mode for all ARTI^a^ diagnoses.

Variables and class				Telehealth		Face-to-face
**Patient characteristics, n (%)**						
	**Patient gender**					
		Men	2034 (39)		7197 (45)	
		Women	3241 (61)		8897 (55)	
	Patient age (year), mean (SD)			32 (20)		25 (22)
	**Patient status, n (%)**					
		Existing patient	1423 (27)		4057 (26)	
		New to registrar	3748 (71)		10277 (64)	
		New to practice	112 (2)		1766 (11)	
**Consultation characteristics, mean (SD)**						
	Consultation duration (min)			12 (6)		16 (7)
	Number of problems managed			1.2 (0.5)		1.3 (0.6)

^a^ARTI: acute respiratory tract infection.

### Main Analyses

Examining each of the 5 individual ARTI conditions: the proportions for which antibiotics were prescribed, and the unadjusted and adjusted ORs (from the univariable and multivariable logistic models) for prescribing antibiotics in telehealth, as compared with F2F consultations, are given in Table 3 (refer to Tables S13-S17 in Multimedia Appendix 5 for the full models). The univariable analyses showed reduced odds of prescribing antibiotics via telehealth (compared with F2F) for sore throat (OR 0.62, 95% CI 0.52-0.74; *P*<.001), and URTI (OR 0.62, 95% CI 0.52-0.75; *P*<.001) but greater odds of prescribing for acute bronchitis (OR 1.40, 95% CI 1.11-1.76; *P*=.005), whilst in multivariable analyses, there were statistically significant differences for sore throat (adjusted OR 0.69, 95% CI 0.55-0.86; *P*=.001), URTI (adjusted OR 0.64, 95% CI 0.51-0.81; *P*<.001), and otitis media (adjusted OR 0.47, 95% CI 0.26-0.84; *P*=.01).

**Table 3:** Univariable and multivariable analyses: antibiotic prescribing for new diagnoses of acute respiratory tract infections during telehealth compared with face-to-face consultations.

Antibiotic prescribed (Yes/No)		All diagnoses (telehealth and face-to-face), n (%)	Diagnoses managed via telehealth, n (%)	Diagnoses managed via face-to-face, n (%)	Uni-variable regression (outcome ‘antibiotics prescribed’): odds ratio for ‘by telehealth’ OR^a^ (95% CI)		*P* value		Adjusted regression: (outcome ‘antibiotics prescribed’): odds ratio for ‘by telehealth’ OR (95% CI)		*P* value	
**Sore throat**						0.62 (0.52-0.74)		<.001		0.69 (0.55-0.86)		.001
	Yes	1685 (51)	261 (41)	1424 (53)								
	No	1642 (49)	380 (59)	1262 (47)								
**URTI^b^**						0.62 (0.52-0.75)		<.001		0.64 (0.51-0.81)		<.001
	Yes	880 (6.9)	178 (4.8)	702 (7.8)								
	No	11,893 (93)	3555 (95)	8338 (92)								
**Sinusitis**						1.10 (0.89-1.38)		.38		1.00 (0.76- 1.32)		.99
	Yes	1067 (61)	294 (62)	773 (60)								
	No	687 (39)	179 (38)	508 (40)								
**Bronchitis**						1.40 (1.11-1.76)		.005		1.07 (0.79-1.45)		.66
	Yes	1140 (64)	255 (70)	885 (63)								
	No	632 (36)	109 (30)	523 (37)								
**Otitis media**						0.69 (0.42-1.14)		.15		0.47 (0.26-0.84)		.01
	Yes	1278 (73)	47 (65)	1231 (73)								
	No	480 (27)	25 (35)	455 (27)								

^a^OR: odds ratio.

^b^URTI: upper respiratory tract infection.

### Post Hoc Analysis

The percentages of registrars’ new ARTI diagnoses with follow-up GP appointments organized are presented in Table 4. There were no statistically significant changes in follow-up, but there was some evidence for more follow-ups following F2F for new otitis media problems (*P*=.06) with clinically significant effect size n/N (11.5%).

**Table 4:** Percentage of follow-up appointments arranged following consultations for new acute respiratory tract infections.

Follow-up arranged (Yes/No)		Telehealth	Face-to-face	*P* value	
**Sore throat, n (%)**					.40
	Yes	259 (40)	1037 (39)		
	No	382 (60)	1649 (61)		
**URTI^a^, n (%)**					.23
	Yes	1087 (29)	2729 (30)		
	No	2646 (71)	6311 (70)		
**Sinusitis, n (%)**					.84
	Yes	152 (32)	418 (33)		
	No	321 (68)	863 (67)		
**Bronchitis, **n** (%)**					.74
	Yes	172 (47)	679 (48)		
	No	192 (53)	729 (52)		
**Otitis media, n (%)**					.06
	Yes	28 (39)	849 (50)		
	No	44 (61)	837 (50)		

^a^URTI: upper respiratory tract infection.

## Discussion

### Principal Findings

On multivariable analysis, antibiotics were (statistically and clinically) significantly more likely to be prescribed in F2F consultations than in telehealth consultations for sore throat, URTI, and otitis media problems. For sinusitis and bronchitis, there were no significant differences in prescribing. There were no significant differences in rates of follow-up consultations organized for ARTIs between the initial consultation modalities, though there was some evidence (*P*=.06) for follow-up appointments to be made more frequently following F2F appointments when the diagnosis was acute otitis media.

### Comparison to Previous Work

A context for our findings (which are specific to antibiotic prescribing for ARTIs) is that previous studies have reported significantly higher rates of prescribing of medicines in general in F2F consultations compared with telehealth consultations, both by Australian GP registrars [34] and in the wider Australian general practice context [35]. In line with this trend, a recent international publication which compared antibiotic prescribing rates for infections (including but not limited to ARTIs) reported higher prescribing rates in F2F consultations [36].

Our results similarly suggest that antibiotics were more likely to be prescribed in F2F consultations for URTI, otitis media, and sore throat. However, these findings differ from results from 2 systematic reviews and meta-analyses [22,23]. Bakhit et al [23] reported greater antibiotic prescribing for otitis media in telehealth consultations, but no difference between consultation modalities in antibiotic prescribing for pharyngitis. Suzuki et al [22] similarly reported that antibiotics were more frequently prescribed via telehealth for otitis media and pharyngitis. This may be partially due to differences in disease classifications. For example, our classification of sore throat includes, but is not limited to, pharyngitis, whereas both of these systematic reviews examine pharyngitis alone, or group all ARTIs together [22,23]. Furthermore, our study was restricted to a GP registrar population, compared with a wider GP population in these systematic reviews.

Regarding sinusitis and bronchitis, our results are consistent with previous studies that reported no significant difference in antibiotic prescribing rates between consultation modalities for sinusitis [22] or bronchitis [23]. In contrast, some studies have reported that fewer antibiotics were prescribed in telehealth vs F2F consultations for sinusitis [23,37,38]. Previous studies reported that the overall antibiotic prescription rates were lower in telehealth consultations for respiratory tract infections [27], especially in early stages of the COVID-19 pandemic (between 2020 2021), both in Australia [26] and internationally [39,40]. Therefore, it is possible that some of these discrepancies may relate to changes in antibiotic prescribing by modality over time, given that our data are from 2020 to 2023.

One possible explanation for our finding of fewer antibiotic prescribing in telehealth consultations is that registrars organized prompt review appointments (eg, to be able to adequately examine the patient) and deferred antibiotic prescription to the F2F review. In a previous qualitative study, some Australian GPs indicated they were not likely to prescribe antibiotics for acute infections without physically examining the patient (in a follow-up consultation) [21]. However, in our post hoc univariate analyses, we found no significant evidence to support this contention. Similarly, a study looking at the management of sinusitis reported no difference in the follow-up rate between e-visits and F2F consultations [38].

Managing patient expectations may be easier in a telehealth consultation compared with a F2F consultation. A recent qualitative study on the management of acute infections via telehealth reported that some GPs found it easier to deny antibiotics during a telehealth consultation, which could be justified by the lack of physical examination [21]. Conversely, other GPs found themselves prescribing more antibiotics in telehealth consultations as a way to mitigate diagnostic uncertainty [21]. Therefore, while it may be easier to manage patient demand via telehealth, the evidence remains mixed.

### Strengths and Limitations

The ReCEnT study has a large sample size, a singularly high response rate for studies of GPs [41], and collects are large amount of registrar, patient and practice variables, which allows for fine-grained adjustment for potential confounding in relationships of factors (eg, consultation mode) with registrars’ prescribing. A particular strength is the tight linkage of prescribed medicine with the diagnosis for which it was prescribed.

A further strength is that, in contrast to a number of other studies of this topic, we examined individual clinical presentations (sore throat, acute bronchitis, acute otitis media, URTI, and acute sinusitis) rather than a heterogeneous combination of conditions, such as “acute respiratory infections” [27].

The generalizability of our registrar population results to the wider, established GP population is potentially limited as vocational training is a singular period in GPs’ professional lives. However, within the apprenticeship-like model of GP training in Australia, registrar prescribing behavior (including antibiotic prescribing) is strongly influenced by, and reflects, the prescribing of their supervisors and senior GP colleagues [20,42]. This is reflected in the temporal pattern of Australian registrars’ antibiotic prescribing mirroring that of established GPs, though at slightly lower rates [15-17]. The generalizability of our findings to vocational training settings in countries beyond Australia is strong in countries with similar apprenticeship-like training structures (eg, the United Kingdom, New Zealand, Ireland, and several European countries). However, generalizability to countries with “residency” training programs, such as in North America [43], is less certain.

This study has several limitations. First, patients’ past medical history and the severity of the index ARTI, which could affect patients and practices electing to schedule telehealth or F2F consultations, were not documented. However, antibiotics are not recommended by authoritative Australian evidence-based guidelines for URTI and acute bronchitis [6], irrespective of severity. For conditions such as acute otitis media, sore throat, and acute sinusitis, where antibiotics are indicated in some circumstances and symptom severity may influence both decision-making and the decisions to consult by F2F or telehealth, symptom severity is an unmeasured potential confounder in our analyses. However, it should be noted that symptom severity is not reliably recorded in primary care databases and is infrequently adjusted for in analyses of antibiotic prescribing [8,23,27,36,44].

Second, ReCEnT does not collect data on whether an antibiotic prescription was filled by the patient. However, this limitation is not of significant importance as the aim of the study was to establish registrars’ prescribing behavior rather than patients’ adherence.

Third, the cross-sectional study design does not allow for implications of causality. However, this study design was appropriate for the aim of estimating the prevalence of antibiotic prescribing by GP registrars for ARTIs during telehealth compared with F2F consultations.

### Future Directions

It is possible that there is more clinical uncertainty in telehealth consultations due to the lack of physical examination, communication barriers, and potential miscommunication from poor internet connection or slow speeds [45]. However, concerns that diagnostic uncertainty (especially due to limitations to physical examination) may drive inappropriate antibiotic prescribing for ARTIs and compromise antimicrobial stewardship have not been supported by our findings, which may, in fact, suggest the contrary. Noting the heterogeneity in the literature on this topic, further research is indicated to establish if our findings are particular to registrars, during the COVID-19 and immediately after the COVID-19 period, or if there is unmeasured confounding in our findings.

Our findings also demonstrate the importance of considering individual respiratory tract infections rather than relying solely on analyses of grouped heterogeneous respiratory infections.

Registrars’ antibiotic prescribing for sore throat, URTI, and otitis media diagnoses was significantly higher in F2F consultation than in telehealth consultations. However, there were no significant differences in antibiotic prescribing for bronchitis or sinusitis between the 2 consultation modalities. Therefore, we have no evidence from our work to discourage registrars from conducting telehealth consultations for ARTIs on the basis of concerns regarding antibiotic stewardship. Further research is needed to explore the reasons why registrars prescribe antibiotics more highly in F2F consultations than telehealth consultations for sore throat, URTI, and otitis media consultations.

## Appendix

Multimedia Appendix 1 ICPC2+ code inclusions for each problem/diagnoses.

Multimedia Appendix 2 Missing data for antibiotic prescribing for each problem/diagnoses.

Multimedia Appendix 3 Most commonly prescribed antibiotics for acute respiratory tract infections by consultation mode.

Multimedia Appendix 4 Characteristics associated with prescribing antibiotics for each problem/diagnoses.

Multimedia Appendix 5 Univariable and multivariable associations with prescribing antibiotics for each problem/diagnoses.

## Abbreviations

ARTI: acute respiratory tract infection
DDD: defined daily doses
F2F: face-to-face
GP: general practitioner
OR: odds ratio
ReCEnT: Registrar Clinical Encounters in Training
URTI: upper respiratory tract infection
